# The Evaluation of Interface Quality in HfO_2_ Films Probed by Time-Dependent Second-Harmonic Generation

**DOI:** 10.3390/ma17143471

**Published:** 2024-07-13

**Authors:** Libo Zhang, Li Ye, Weiwei Zhao, Chongji Huang, Xue Liu, Wenshuai Gao, Tao Li, Tai Min, Jinbo Yang, Mingliang Tian, Xuegang Chen

**Affiliations:** 1 Center of Free Electron Laser & High Magnetic Field, Leibniz International Joint Research Center of Materials Sciences of Anhui Province, Anhui University, Hefei 230601, China; 2 School of Materials Science and Engineering, Anhui University, Hefei 230601, China; 3 Shanghai Aspiring Semiconductor Equipment Co., Ltd. & Aspiring Semiconductor (Beijing) Co., Ltd., Shanghai 200082, China; 4 Center for Spintronics and Quantum Systems, State Key Laboratory for Mechanical Behavior of Materials, Department of Materials Science and Engineering, Xi’an Jiaotong University, Xi’an 710049, China; 5 State Key Laboratory for Mesoscopic Physics, School of Physics, Peking University, Beijing 100871, China; 6 School of Physics and Optoelectronic Engineering, Anhui University, Hefei 230601, China; 7 Anhui Province Key Laboratory of Condensed Matter Physics at Extreme Conditions, High Magnetic Field Laboratory, Chinese Academy of Sciences, Hefei 230031, China; 8 Information Materials and Intelligent Sensing Laboratory of Anhui Province, Anhui Key Laboratory of Magnetic Functional Materials and Devices, Anhui University, Hefei 230601, China

**Keywords:** time-dependent second-harmonic generation, HfO_2_ film, fixed charge density, interface state density, capacitance–voltage/conductance–voltage

## Abstract

Time-dependent second-harmonic generation (TD-SHG) is an emerging sensitive and fast method to qualitatively evaluate the interface quality of the oxide/Si heterostructures, which is closely related to the interfacial electric field. Here, the TD-SHG is used to explore the interface quality of atomic layer deposited HfO_2_ films on Si substrates. The critical SHG parameters, such as the initial SHG signal and characteristic time constant, are compared with the fixed charge density ($Q_{ox}$) and the interface state density ($D_{it}$) extracted from the conventional electrical characterization method. It reveals that the initial SHG signal linearly decreases with the increase in $Q_{ox}$, while $D_{it}$ is linearly correlated to the characteristic time constant. It verifies that the TD-SHG is a sensitive and fast method, as well as simple and noncontact, for evaluating the interface quality of oxide/Si heterostructures, which may facilitate the in-line semiconductor test.

## 1. Introduction

To meet the requirements of semiconductor device integration, the size of metal-oxide-semiconductor field effect transistor (MOSFET) continues to shrink, approaching the physical limitation [1,2]. A key issue is that the performance of the MOSFET is closely related to the quality of the interface between the semiconductor and the oxide layer [3,4,5,6]. Although the traditional electrical characterization methods, such as voltage–capacitance method (C–V) [7,8,9], conductance method (G–V) [10], Terman method [11], etc., can accurately identify the interface quality, they are invasive (requiring preparation of specific electronic devices) [2,7,12,13,14,15], resulting in irreversible damage to devices or wafers, and the characterization is unable to provide real-time feedback [16]. Alternatively, the optical characterization is an efficient and noninvasive method to detect the interface quality, which may have great potential application in the in-line test during the functional device fabrications [17,18].

Since van Driel revealed the ability of time-dependent second-harmonic generation (TD-SHG) to detect the interfacial bonding in the Si wafer [17], the TD-SHG technique has been widely explored for disclosing the charge trapping/detrapping as well as the carrier transport properties at the interface [19,20,21,22]. Recently, the noncontact TD-SHG method was reported to characterize the charge trapping in high-k dielectric structures, considering the inversion symmetry breaking at the interface [21,23,24,25]. Generally, the separation of carriers at the interface due to the laser illumination induces a quasi-static interfacial electric field E(t), which determines the intensity of second-harmonic signal ($I_{2\omega }$). It can be expressed by the following equation [26,27]:

(1) $$I_{2\omega }(t)\propto {\left|\chi_{interface}^{\left(2\right)}+\chi^{\left(3\right)}\left|\;[E_{dc}+E(t)]\right|\right|}^{2}I_{\omega }^{2}$$

Here, $I_{2\omega }(t)$, ${\;\chi }_{interface}^{2}$, $\chi^{3}$, and $I_{\omega }$ are the intensity of SHG, the second-order nonlinear susceptibility at the interface, the third-order nonlinear susceptibility, and the intensity of incident laser, respectively. In addition, the TD-SHG was used to study the dopant type and dopant density of the materials as well as the fixed charges. Although various studies have been conducted, there is a lack of comprehensive study of the correlation between the signal of SHG and the interface quality [25,28,29].

In this study, the atomic layer deposited HfO_2_/n-Si films were chosen as a protype to investigate the correlation between TD-SHG and interface quality of oxide/Si heterostructures. The HfO_2_ films display a good insulating character with a low leaky current with an applied voltage. It was found that the TD-SHG monotonically increases with the evolution of time. It reveals that the initial SHG signal linearly decreases with the increase in fixed charge density ($Q_{ox}$), while the interface state density ($D_{it}$) is linearly correlated to the characteristic time constant. It indicates that the TD-SHG technique is a sensitive and fast method for assessing the interface quality of oxide/Si heterostructures, which provides an effective means for online interface quality detection.

## 2. Materials and Methods

Various thicknesses of HfO_2_ thin films (5–20 nm) were deposited on the n-type Si(001) substrates (resistivities of 1–30 Ω·cm) via the atomic layer deposition technique. The square metal electrodes (Au (80 nm)/Ti (5 nm)) with different pad sizes were prepared by the conventional photolithography, followed by the e-beam evaporation process. The crystalline structure of HfO_2_ thin films was investigated by the X-ray diffraction (XRD, model D8 ADVANCE, Bruker, Germany). The surface morphology of the HfO_2_ film was measured by atomic force microscopy (AFM, model AFM5500M, Tokyo, Japan). The J–V characteristics were tested in a home-build setup with a Keithley (Cleveland, OH, USA) 2636B source measure meter controlled by a program. The C–V and G–V measurements were carried out using a Keysight (Santa Rosa, Ca, USA) E4980A precision LCR meter. The positive voltage is always defined as a voltage applied on the top Au electrode. The TD-SHG was performed using an Aspirer 3000 system (Beijing, China) with the laser of 780 nm (repetition frequency 80 MHz, pulse width 150 fs). The incident P-polarized laser (780 nm) illuminates on the sample at 45°. The generated second-harmonic signal (λ = 390 nm) was collected. The TD-SHG experiment was performed after the maximum direction of the SHG pattern was determined according to the rotation-anisotropy SHG results to provide a standard process of charge evolution. All the TD-SHG measurements in our experiments were conducted at room temperature with a dark environment.

## 3. Results

**Figure 1:**
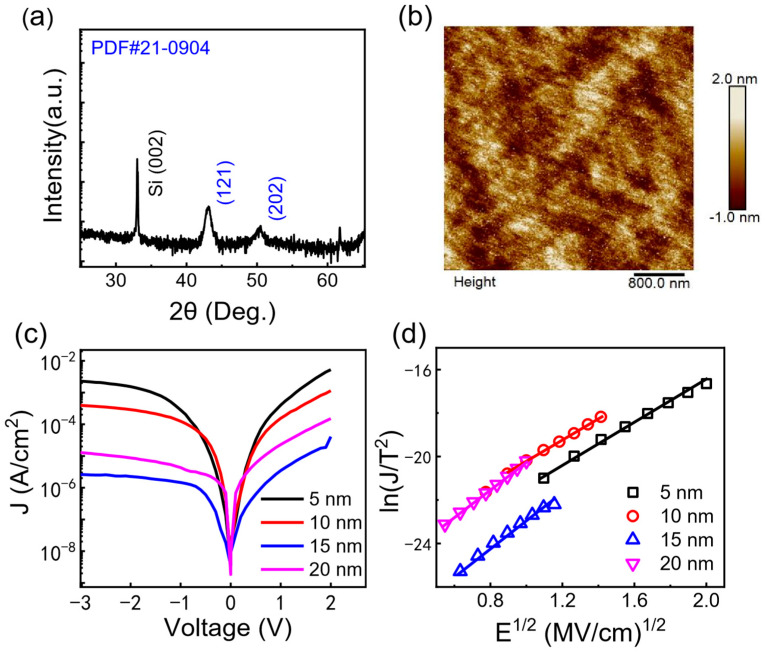
(**a**) The typical XRD pattern of as-deposited HfO_2_ film on Si substrate. (**b**) The typical atomic force microscopy image of 20 nm HfO_2_ film. (**c**) The current density vs. the applied voltage (J–V curve) for various thickness of HfO_2_ films. (**d**) The Schottky emission (SE) fitting of J–V curve.

Figure 1a displays the typical XRD pattern of the HfO_2_ film grown on a Si substrate. Clear diffraction peaks from HfO_2_ film located at 43.2° (121) and 50.4° (202) are observed. Figure 1b shows the typical atomic force microscopy image of the HfO_2_ film (20 nm), which is scanned over the area of 4 μm × 4 μm. The surface roughness of the as-deposited HfO_2_ thin film is 0.43 nm, indicating the uniform and smooth surface of the HfO_2_ film. The current–voltage curves (J–V) of the samples with different thickness measured at room temperature are shown in Figure 1c. Clearly, the HfO_2_ films reveal a low current density (~nA level), indicating a high quality of HfO_2_ film. The current–voltage relation can be well characterized by the Schottky emission (SE) [30,31,32]:

(2) $$J_{SE}=A^{*}T^{2}\exp\left[\frac{-q\left(\varphi_{B}-\sqrt{qE/4\pi \varepsilon_{0}\varepsilon_{r}}\right)}{k_{B}T}\right]\;$$

Here, $A^{*}$, *T*, $k_{B}$, E, $\varphi_{B}$, $\varepsilon_{0}$, and $\varepsilon_{r}$ are the Richardson constant 120 A/(cm^2^·K^2^), the absolute temperature, the Boltzmann constant, the electric field, the Schottky barrier height, the vacuum dielectric constant, and the relative dielectric constants, respectively. The current can be well fitted by the SE, relation as shown in Figure 1d. The extracted Schottky barrier heights are around 0.80 eV, irrespective of the HfO_2_ thickness (Table 1), verifying the high quality of the HfO_2_ film.

**Figure 2:**
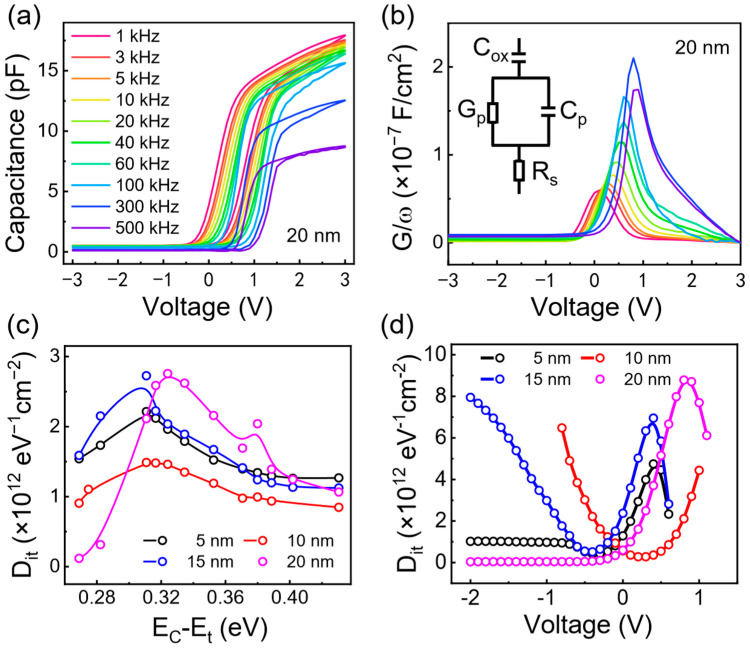
(**a**) The corrected C–V curves for typical 20 nm HfO_2_ film with various frequency ranging from 1 kHz to 500 kHz. (**b**) The corrected G–V curves for typical 20 nm HfO_2_ film. (**c**) The relation between the extracted $D_{it}$ and the energy level ($E_{C}-E_{t}$), and (**d**) the applied voltage dependent of $D_{it}$ with various thickness of HfO_2_ films.

In order to reveal the interface quality of the HfO_2_/Si interface, the conventional electrical characterization with the metal electrodes was conducted. The series resistance correction (SRC) model is used to correct the measured capacitance–voltage (C–V) and conductance–voltage (G–V) [33,34,35,36]. The corrected C–V and G–V are displayed in Figure 2. A clear C–V hysteresis is observed at the positive bias, corroborating the existence of the border traps near the interface. The capacitance at +3 V does not saturate at the accumulation region, indicating the existence of carrier trapping. The extracted fixed charge ($Q_{ox}$) increases from 1.43 × 10^11^ cm^−2^ (5 nm) to 2.74 × 10^11^ cm^−2^ (15 nm) (Table 1) [26,30]. Generally, a conductance peak appears when sweeping the frequency at a certain voltage, corresponding to the maximum energy loss due to the interface traps resonation. Clearly, the G/ω peak moves to the high-voltage position with the increase in frequency, accompanying the increase in the peak magnitude. Therefore, the interface state density can be quantitively calculated by the relation $D_{it}\approx \frac{2.5}{Aq}{\left(\frac{G_{P}}{\omega }\right)}_{max}$, where A and q are the electrode area (50 μm × 50 μm) and the element charge. Additionally, the distribution of $D_{it}$ as a function of energetic position (ΔE) in the upper region of Si band gap can be roughly estimated using the full interface state model. The energetic position is the energy difference between the trap energy level ($E_{t}$) and the majority carrier band edge energy level ($E_{C}$ or $E_{V}$), which can be calculated by the following equation [31]:

(3) $$∆E=E_{C}-E_{t}=\frac{k_{B}T}{q}\times \ln\left(\frac{\sigma v_{th}D_{dos}}{\omega }\right)$$

Here, ∆E is the difference between the trap energy level ($E_{t}$) and the majority carrier band edge energy level ($E_{C}$ or $E_{V}$). σ, $v_{th}$, and $D_{dos}$, are the trap capture cross-section (1.0 × 10^15^ cm^−2^), the average hot carrier rate (1.6 × 10^7^ cm·s^−1^), and the effective conduction band density of states (2.8 × 10^19^ cm^−3^) [37]. The extracted ∆E and $D_{it}$ are displayed in Figure 2c, which reveals a comparatively low $D_{it}$ near the Si conduction band/far from the Si conduction band. A $D_{it}$ peak is found at around 0.31 eV regardless of HfO_2_ thickness, namely, the $D_{it}$ values of 3.09 × 10^12^ eV^−1^cm^−2^ (5 nm), 2.08 × 10^12^ eV^−1^cm^−2^ (10 nm), 3.81 × 10^12^ eV^−1^cm^−2^ (15 nm), and 4.39 × 10^12^ eV^−1^cm^−2^ (20 nm). In addition, the applied voltage dependent $D_{it}$ is displayed in Figure 2d. The values of $D_{it}$ near the flat band voltage are 4.03 × 10^12^ eV^−1^cm^−2^ (5 nm), 2.89 × 10^12^ eV^−1^cm^−2^ (10 nm), 4.52 × 10^12^ eV^−1^cm^−2^ (15 nm), and 5.26 × 10^12^ eV^−1^cm^−2^ (20 nm). It seems that there is a correlation between the voltage of $D_{it}$ peak position and flat band voltage, which need to be explored in future. Additionally, the interface state density obtained from both conductivity and capacitance methods show a consistent trend, indicating that the HfO_2_/Si interface is a good protype for the TD-SHG study.

**Figure 3:**
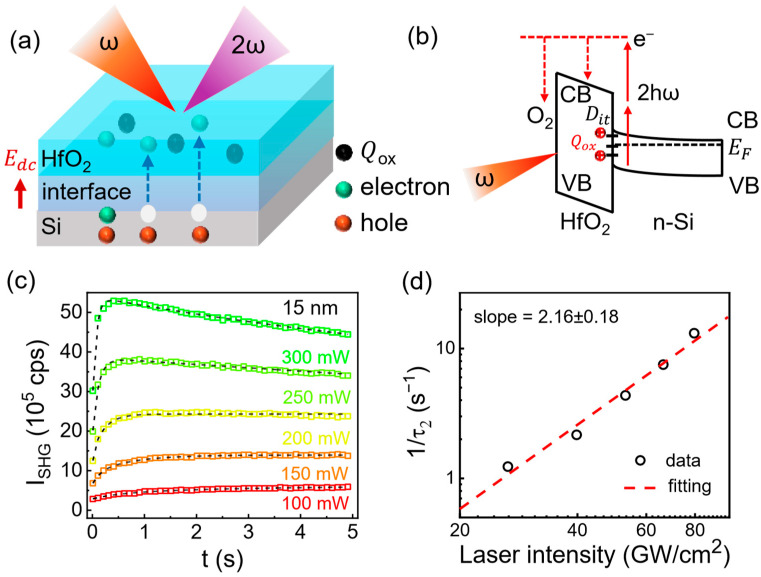
(**a**) The schematic of second-harmonic generation for HfO_2_/Si. (**b**) The corresponding schematic energy diagram. (**c**) The TD-SHG signals under different laser power for a typical 15 nm HfO_2_ film. The corresponding fitting lines are shown in black. (**d**) The laser intensity dependence of extracted time constant $\tau_{2}$.

Generally, the time-dependent second-harmonic generation (TD-SHG) signal can be used to comprehensively understand the laser-induced electron transport dynamics in the oxide/Si systems [38]. A schematic of laser-induced electron transport/transfer, as well as the generation of SHG, is displayed in Figure 3a. In this case, the internal electric field $E_{dc}$ forms due to the existence of the fixed charges before the laser illumination, corresponding to the SHG signal at the initial state. After the laser illumination, electrons in Si are excited/transferred into the HfO_2_ film, while the holes remain in Si. Correspondingly, the laser-induced electric field contributes to the SHG signal. Continuous laser illumination could generate photoexcited electrons that become trapped at the border and interface trap states, dominating the interfacial electric field and SHG signal. In this scenario, the TD-SHG is used to effectively identify the time evolution of the interfacial electric field, which can be closely correlated to the interface traps. In the HfO_2_/Si system, the interfacial electric field arises from the laser-induced multiphoton excitation (Figure 3b). Figure 3c displays the laser power dependency of TD-SHG. The TD-SHG with a low power (≤150 mW) shows a monotonically increase in SHG signal, which tends to saturate in a short time. It indicates that the interface electric field increases with the continued increase in laser irradiation, and, subsequently, the laser-induced captured electrons reach a balance with the recombination of electrons and holes at the interface. The SHG signal is significantly enhanced with the increase in laser power, considering the greatly increased possibility of more electrons excitation under high laser power. When a laser with a power of 300 mW irradiates on the 15 nm HfO_2_/Si sample, the SHG signal rises quickly (<0.5 s), following a slight decay with the evolution of time. This may be related to the transfer process of electrons from the oxide back to the Si substrate, resulting in the subsequent decay SHG signal.

The collected TD-SHG data can be well fitted by the following equation [20,26,39]:

(4) $$\sqrt{I_{2\omega }(t)}\propto \chi_{interface}^{\left(2\right)}+{\chi^{\left(3\right)}E}_{0}e^{-\frac{t}{\tau_{1}}}-{\chi^{\left(3\right)}E}_{1}\left(1-e^{-\frac{t}{\tau_{2}}}\right)$$

where $E_{0}$ and $E_{1}$ are the electric field induced by the fixed charge $Q_{OX}$ and the electric field induced by interface charge traps, respectively. ${\;\chi }_{interface}^{2}$, $\chi^{3}$, $\tau_{1}$, and $\tau_{2}$ are the second-order nonlinear susceptibility at the interface, the third-order nonlinear susceptibility, and the trapping time constant ($\tau_{i}$) corresponding to the fast ($\tau_{1}$) and slow ($\tau_{2}$) trapping process. This equation is sufficient to depict the dynamic process of the laser-induced interfacial electric field. The exacted $1/\tau_{2}$ under various laser power is displayed in Figure 3d. The electron trapping rate $1/\tau_{2}$ linearly increases with the increase in power density, which yields the relation $1/\tau_{2}\propto {\left(I_{\omega }\right)}^{n}$ (n represents the number of photons involved in multiphoton absorption) [40,41]. Here, the fitted n is 2.16 ± 0.18, indicating that a two-photon absorption is needed to excite the electrons from the valence band (VB) of Si to the conduction band (CB) of HfO_2_. It is consistent with the laser excitation energy of 1.59 eV (780 nm) and band offset 3.14–3.72 eV between the Si and HfO_2_, namely, the two-photon excitation process.

**Figure 4:**
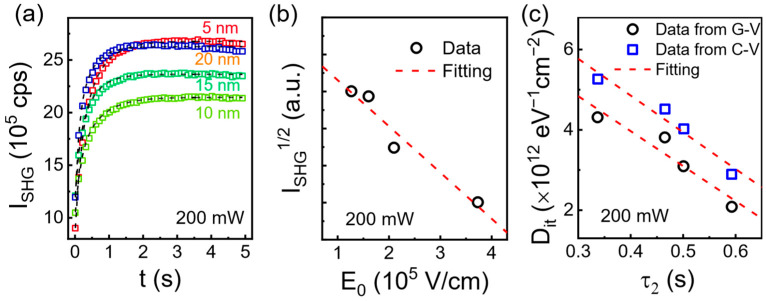
(**a**) The TD-SHG signal under 200 mW for various thickness of HfO_2_ films. (**b**) The relation between the electric field from the fixed charge density from C–V and the initial SHG intensity. (**c**) The relation between the extracted time constant $\tau_{2}$ from TD-SHG and the extracted interface state density from C–V/G–V.

In order to evaluate the ability of TD-SHG to reveal the quality of the oxide/semiconductor interface, the relation between the critical time constant of TD-SHG and the fixed charge density/interface state density was studied. Figure 4a displays the typical TD-SHG signal with the laser illumination power of 200 mW for various thickness of HfO_2_ films (5–20 nm). Obviously, the TD-SHG shows a monotonical increase with the time. A fast increase in SHG signal in ~1 s is followed by a slow saturation in 5 s. The saturated SHG signal increases with the HfO_2_ thickness except for the 5 nm film, considering that the electrons can easily transfer/tunnel through the thin HfO_2_ film. The initial point of the SHG signal increases with the HfO_2_ thickness. Commonly, the initial interfacial electric field $E_{0}$ is closely related to the fixed charge density $Q_{ox}$ (calculated from the conventional C–V method) through the Gauss relation $E_{Q_{ox}}=Q_{ox}/(\varepsilon_{Si}\times q)$, where $\varepsilon_{Si}$ and q are the dielectric constant of Si and the element charge, respectively. It is natural to connect the initial SHG signal with the initial interfacial electric field, namely, the fixed charge density. Accordingly, the initial interfacial electric field dependent on the square root of SHG signal is plotted in Figure 4b. A linear relation is revealed between $E_{Q_{ox}}$ and $\sqrt{I_{SHG}}$, indicating that it can be used to explain the observed phenomenon. The substrate used in the experiment is n-type silicon substrate (resistivity of 1–30 Ω·cm), and, as such, the fixed charge density is lower than the ionized donor density; hence, a larger $Q_{ox}$ density will result in a smaller initial SHG intensity. It confirms that the TD-SHG can be efficiently used to evaluate the fixed charge density in the HfO_2_/Si films.

The TD-SHG is an emerging method used to evaluate the quality of a semiconductor, which is closely related to the electron dynamics including the electron excitation, transport, and trapping/detrapping. In this scenario, the laser irradiation could generate a time-dependent quasistatic electric field, which can be significantly affected by the interface state density considering the dynamic process. Therefore, the characteristic parameter $\tau_{2}$ can be connected to the interface state density. The characteristic parameter $\tau_{2}$ is extracted for various thickness of HfO_2_ films according to Equation (4). Figure 4c displays the relation between the extracted $\tau_{2}$ and the calculated $D_{it}$ (conventional C–V and G–V methods). Clearly, the linear relation between $D_{it}$ and $\tau_{2}$ is revealed. A small $\tau_{2}$ means a fast trapping/detrapping rate to reach a balance, corresponding to a large interface state density at the interface. The experimental results verify that the TD-SHG is a simple and fast method for extracting the important semiconductor parameters such as $Q_{ox}$, $D_{it}$, etc., which may facilitate the in-line semiconductor monitoring.

## 4. Conclusions

In this study, the TD-SHG method was employed to qualitatively characterize the interface states in the HfO_2_/Si films, which are compared with the traditional electrical methods. The electric-field-induced SHG signal indicates that the initial SHG intensity correlates with the electrostatic field strength induced by fixed charges in the oxide layer, as revealed by conventional C–V measurements. Furthermore, the evolution of the SHG signal over time varies with the $D_{it}$ extracted from C–V and G–V measurements. The higher $D_{it}$ is associated with a fast SHG evolution, while the lower value corresponds to a slow SHG evolution. This confirms the feasibility of using SHG to probe the quality of the HfO_2_/Si interface. This study validates that TD-SHG is a sensitive and rapid method to assess the interface quality in the oxide/Si heterojunctions, which could be beneficial for in-line testing in semiconductor fabrication.

## Figures and Tables

**Table 1:** The HfO_2_ thickness dependent of extracted parameters including the flat band voltage, ${q\varphi }_{B}$, the $Q_{ox}$, and the $D_{it}$.

Sample	${q\varphi }_{B}$ (V)	$V_{fb}$ (V)	$Q_{ox}$ (×10^11^ cm^−2^)	$D_{it}$ (×10^12^ eV^−1^cm^−2^)
5 nm	0.80	0.43	1.43	3.09
10 nm	0.79	0.60	1.96	2.08
15 nm	0.88	0.51	2.74	3.81
20 nm	−0.82	0.63	2.39	4.31

## Data Availability

The original contributions presented in the study are included in the article, further inquiries can be directed to the corresponding author.
